# MicroRNAs in rhabdomyosarcoma: pathogenetic implications and translational potentiality

**DOI:** 10.1186/1476-4598-10-120

**Published:** 2011-09-24

**Authors:** Rossella Rota, Roberta Ciarapica, Antonio Giordano, Lucio Miele, Franco Locatelli

**Affiliations:** 1 Department of Oncohematology, Ospedale Pediatrico Bambino Gesù, IRCCS, Roma, Italy; 2 Sbarro Institute for Cancer Research and Molecular Medicine and Center of Biotechnology, Temple University, Philadelphia, PA, USA; 3 Department of Human Pathology and Oncology, Università di Siena, Siena, Italy; 4 Cancer Institute, University of Mississippi Medical Center, Jackson, MS, USA; 5 Dipartimento di Scienze Pediatriche, Università di Pavia, Pavia, Italy

## Abstract

There is growing evidence that interconnections among molecular pathways governing tissue differentiation are nodal points for malignant transformation. In this scenario, microRNAs appear as crucial players. This class of non-coding small regulatory RNA molecules controls developmental programs by modulating gene expression through post-transcriptional silencing of target mRNAs. During myogenesis, muscle-specific and ubiquitously-expressed microRNAs tightly control muscle tissue differentiation. In recent years, microRNAs have emerged as prominent players in cancer as well. Rhabdomyosarcoma is a pediatric skeletal muscle-derived soft-tissue sarcoma that originates from myogenic precursors arrested at different stages of differentiation and that continue to proliferate indefinitely. MicroRNAs involved in muscle cell fate determination appear down-regulated in rhabdomyosarcoma primary tumors and cell lines compared to their normal counterparts. More importantly, they behave as tumor suppressors in this malignancy, as their re-expression is sufficient to restore the differentiation capability of tumor cells and to prevent tumor growth *in vivo*. In addition, up-regulation of pro-oncogenic microRNAs has also been recently detected in rhabdomyosarcoma.

In this review, we provide an overview of current knowledge on microRNAs de-regulation in rhabdomyosarcoma. Additionally, we examine the potential of microRNAs as prognostic and diagnostic markers in this soft-tissue sarcoma, and discuss possible therapeutic applications and challenges of a "microRNA therapy".

## Introduction

Since the discovery of the function of lin-4, the first discovered canonical microRNA (miRNA) in *Caenorhabditis elegans *[1-3], more than 1400 miRNAs have been identified in mammals (miRBASE, http://www.mirbase.org), most of which with unknown functions.

Mature miRNAs are a class of non-coding ~ 19-25 nucleotide (nt) single-strand RNAs highly conserved across species. They act by binding complementary sequences in the 3'-untranslated regions (UTRs) of a messenger RNA (mRNA) through their "seed" sequence (nt 2-8 at the 5' end), with either incomplete or complete base-pairing. This leads to either translational repression or transcriptional degradation of target mRNAs [4]. The end result is post-transcriptional silencing of selected genes that provides an additional layer of gene expression control and enhances the flexibility of gene regulation. A relatively low stringency requirement for base pairing between a particular miRNA and its target 3'UTR sequences results in the capacity of each miRNA to silence several mRNAs [5]. Consequently, small changes in miRNAs expression can have significant effects on cellular phenotype. Conversely, the same mRNA can be targeted by several miRNAs.

**Figure 1:**
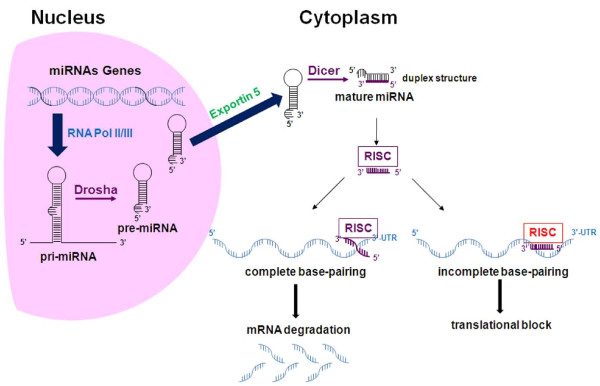
**Schematic representation of biogenesis and function of miRNAs**. miRNAs genes, in mono- (as reported in the picture) or polycistronic structures are transcribed by RNA Polymerases II and III as several kb long transcripts (pri-miRNA) characterized by a hairpin structure, and then cleaved by the nuclear RNAse III Drosha. These miRNAs precursors (pre-miRNA) are carried by the Exportin 5 to the cytoplasm where they are subjected to further digestion by the cytoplasmic RNAse III Dicer giving rise to a duplex 19-25 nt microRNA structure. One strand (guided strand) of the duplex (mature miRNA) is incorporated into the RISC complex and carried to the 3'UTR of target mRNAs. If the complementarity between the seed sequence of a miRNA and the 3'UTR mRNA sequence is 100% the mRNA is degraded while suppression of translation is obtained for lower degrees of complementarities.

Genes encoding for miRNAs are evolutionarily conserved and the majority of these are located in intergenic regions or in antisense orientation, suggesting that they behave as independent transcription units. Other miRNAs can be present in intronic regions and transcribed as part of annotated genes. miRNAs can form clusters transcribed as polycistronic transcripts by RNA polymerase II and/or III [6,7], which undergo sequential steps of maturation (Figure 1) [4,8]. The first step is catalyzed within the nucleus by RNase III Drosha that generates pre-miRNAs molecules of ~ 70 nt. After shuttling to the cytoplasm, pre-miRNAs are further processed by RNase III Dicer. The result is a 19 to 25-nt double-strand RNA. Of the 2 RNA strands the less stable one is the mature miRNA, which is incorporated into the RNA-induced silencing complex (RISC). The RISC is necessary for the annealing of miRNAs to the 3'UTR regions of target mRNAs [4,9]. The complementary strand, which is generally indicated with an asterisk, is released and generally, though not always, degraded [10]. The expression of mature miRNAs is subjected to stringent transcriptional and post-transcriptional regulation.

A connection between miRNAs and differentiative processes emerged initially from studies on worms and Drosophila, where miRNAs play fundamental roles in developmental timing and tissue differentiation [3,11]. Subsequent loss-of-function studies on miRNAs, or on proteins responsible for miRNA maturation, confirmed that these small RNAs are crucial regulators of development, stem cell fate and maintenance of tissue identity in vertebrates as well [12-16].

In mammals, miRNAs participate in the organization of tissue and organ diversity during the embryonal life. Physiologically, the majority of miRNAs function in a tissue-specific manner, by preventing the expression of genes that should not be expressed in a particular tissue context and the inappropriate expansion of tissue precursors. To date, miRNAs have been involved in a considerable number of physiologic and pathologic processes such as aging, cancer, metastasis, angiogenesis and immune regulation [17-19]. De-regulation of miRNAs expression in cancer was first reported for chronic lymphocytic leukemia [20]. Although some miRNAs can act as oncogenes, miRNAs identified as de-regulated in cancer are more commonly tumor suppressors. These tumor suppressor miRNAs are globally down-regulated by several mechanisms involving rearrangements of unstable chromosomal regions, mutations or epigenetic silencing [21-23]. Several studies have suggested that normalization of miRNAs expression could be used as a differentiation therapy in cancer [24-29].

Rhabdomyosarcoma (RMS), the most common soft-tissue sarcoma of childhood, is an attractive target for differentiation therapy [30]. RMS is a skeletal muscle-derived tumor widely thought to be originated from myogenic precursors unable to differentiate [31]. It consistently expresses muscle-specific transcription factors such as Myogenic Differentiation (MyoD) and myogenin but shows no sign of terminal muscle differentiation [32]. Therefore, strategies aimed at restoring the myogenic program reverse RMS cell malignant behavior and are a conceptually acceptable therapeutic intervention.

RMS accounts for approximately 6-8% of all pediatric tumors; it includes two major histological subtypes, namely embryonal and alveolar RMS [33,34]. The former has a better prognosis, the 5-year overall survival rate of patients with this histological variant being 70% or even more. Alveolar RMS accounts for about 25% of RMS but predicts a poorer outcome. In around 75-80% of cases, alveolar RMS is characterized by recurrent chromosomal translocations. The more common are the t(2;13) or t(1;13) that result in the expression of the oncogenic fusion proteins PAX3-FKHR or PAX7-FKHR [35-38]. The remaining 20-25% of alveolar RMS forms are considered fusion-negative, *i.e*., they do not express any known fusion protein. Clinical-pathological risk factors have been largely used for patient risk stratification at diagnosis. In particular, alveolar histology and metastasis represent the most important poor-prognosis variables, predicting a dismal outcome. The detection of oncogenic fusion proteins, and especially PAX3-FKHR, in alveolar RMS have a clear prognostic value, as they characterize a distinctly aggressive subgroup frequently unresponsive to conventional therapies and with a high risk of recurrence [36,39]. The correlation between the presence of oncogenic fusion proteins and poor prognosis has been recently corroborated by gene expression profiling studies indicating that fusion-positivity is a risk factor independent from histology [40,41]. Indeed, the same studies showed that fusion-negative alveolar tumors have gene expression patterns similar to that of embryonal RMSs. Previously published studies on gene expression and immunohistochemical analyses suggested that alveolar fusion-positive and the majority of embryonal RMS are two distinct groups also according to the level of expression of two specific sets of genes [37,42]. One of these studies also demonstrated that fusion-negative alveolar and a small portion of embryonal tumors were characterized by intermediate expression levels of specific genes and were difficult to be clearly distinguished from each other [42]. Since about 50% of RMSs, including the majority of alveolar fusion-negative tumors, are intermediate-risk forms, a clearer sub-classification of these tumors may greatly improve clinical management [43]. In this regard, atypical chromosomal translocations have been recently reported in fusion-negative alveolar tumors. These previously undetected cytogenetic anomalies could, at least partly, explain the molecular and clinical heterogeneity found in RMS [44].

Recently, some miRNAs acting as key regulators of skeletal muscle cell fate determination have been shown to be de-regulated in both alveolar and embryonal RMS. Gain-of-function experiments have demonstrated that re-expression of selected "tumor-suppressor" miRNAs impairs the tumorigenic behavior of RMS cells. Moreover, miRNA expression profiling appears to be a promising strategy for discriminating specific variants among RMS subsets and for providing useful prognostic information, especially for what concerns fusion-negative alveolar and embryonal forms [45]. These observations suggest that miRNA de-regulation may be involved in the pathogenesis of RMS. Additionally, the expression of miRNAs with pro-oncogenic properties has been reported in RMS.

In this article, we review our current knowledge on de-regulation of miRNAs in RMS. We also examine the potentiality of these small RNAs as diagnostic and/or prognostic biomarkers. Finally, we discuss the implications and challenges of a potential "miRNA therapy" in RMS.

### Regulation and function of miRNAs in skeletal muscle differentiation

An exhaustive report on the regulation of skeletal muscle differentiation by miRNAs is outside the scope of this manuscript. However, to understand the complexity of miRNAs molecular networks correlated to RMS pathogenesis, we summarize current knowledge on the physiologic regulation and function of selected miRNAs.

Embryonic mesoderm gives rise to cardiac, skeletal and smooth muscle tissues. During skeletal muscle tissue differentiation, cell precursors proliferate, migrate to specific tissue sites, elongate and fuse to each other forming multinucleated myotubes. The differentiation of stem cells into skeletal muscle tissue occurs through a tightly controlled spatial and temporal molecular cascade that involves miRNAs. The importance of these non-coding regulatory small RNAs in myogenesis has been recently highlighted by studies on mice conditionally deleted in a Dicer allele in skeletal muscle progenitors. These mice show severe muscle hypoplasia associated with perinatal death [46]. miRNAs involved in myogenesis include both muscle-specific miRNAs, which are selectively expressed in muscle tissues, and miRNAs that are ubiquitously expressed but play a role in the myogenic process.

#### Muscle-specific miRNAs in skeletal muscle differentiation

**Figure 2:**
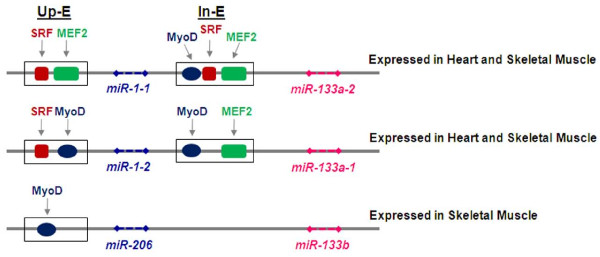
**Schematic overview of three myomiR clusters**. Three bicistronic myomiR clusters and cis-regulatory elements are shown. Myogenic regulatory factors SRF, MEF2 and MyoD bind to an upstream enhancer (Up-E) and/or intronic enhancer (In-E) to transactivate miRNA transcription. miR-1-1/miR-133a-2 and miR-1-2/miR-133a-1 are induced in both cardiac and skeletal muscle while miR-206/miR-133b is skeletal muscle specifically expressed.

miRNAs that specifically control cell fate determination of myogenic precursors and muscle tissue homeostasis are referred to as "myomiRs" [47]. MyomiRs include the miR-1/miR-206 family, encoded by 3 bicistronic miRNA gene clusters on 3 separate chromosomes: miR-1-1/miR-133a-2, miR-1-2/miR133a-1 and miR-206/miR-133b (Figure 2). miR-1-1 and miR-1-2 are involved in both cardiac and skeletal muscle development and have identical nucleotide sequences, while miR-206 is specifically expressed in skeletal muscle and differs in 4 nucleotides outside the seed sequence (Table 1) [48-52]. miR-133a-1 and miR-133a-2 are identical in sequence, differing from miR-133b by a single nucleotide (Table 1) [49]. Another member of myomiRs is the miR-208, specifically expressed in heart [53]. It is worth noting that myomiRs are naturally able to specify and maintain the muscle identity of a tissue because forced expression of miR-1 in epithelioid HeLa cells or in embryonic stem cells represses non-muscle genes, while inducing an expression profile reminiscent of muscle cells [54,55]. The transcription factor Serum Response Factor (SRF) and myogenic regulatory factors (MRFs) such as Myocyte Enhancer Factor 2 (MEF2), MyoD and myogenin [56] regulate myomiR expression during muscle tissue differentiation by binding specific promoter and/or enhancer sites on target miRNAs genes.

**Table 1:** Characteristics of de-regulated miRNAs in rhabdomyosarcoma


**myomiRs**	Sequence*	**Human chromosome**	**Region**

miR-1-1	5'-U**GGAAUGU**AAAGAAGUAUGUA-3'	20	Intergenic
miR-1-2	5'-U**GGAAUGU**AAAGAAGUAUGUA-3'	18	Intronic (*Mindbomb*)
miR-206	5'-U**GGAAUGU**AAGGAAGUGUGUGG-3'	6	Intergenic

miR-133a-2	5'-U**UUGGUCC**CCUUCAACCAGCUG-3'	20	Intronic
miR-133a-1	5'-U**UUGGUCC**CCUUCAACCAGCUG-3'	18	Intronic (*Mindbomb*)
miR-133b	5'-U**UUGGUCC**CCUUCAACCAGCUA-3'	6	Intronic

**Non-muscle miRNAs**			

miR-29b2	5'-U**AGCACCA**UUUGAAAUCAGUGUU-3'	1	Intergenic
miR-29c	5'-U**AGCACCA**UUUGAAAUCGGGUUA-3'	1	Intergenic

miR-26a	5'-U**UCAAGUA**AUCCAGGAUAGGC-3'	3	Intronic (*CTDSPL*)

miR-183	5'-U**AUGGCAC**UGGUAGAAUUCACU-3'	7	Intergenic

miR-17	5'-C**AAAGUGC**UUACAGUGCAGGUAG-3'	13	Intronic

*Seed sequences in **bold **(from TargetScan: www.targetscan.org). Nucleotide differences among myomiRs gene families (grouped) are underlined.

SRF and MRFs cooperate during differentiation in cardiac and striated muscle, by inducing the expression of miR-1-1/miR-133a-2 and miR-1-2/miR133a-1 (Figure 2) [57,58]. Instead, the miR-206/miR-133b cluster is induced by MyoD and myogenin during the early phases of skeletal myogenic differentiation [50,52,59,60]. Interestingly, miR-1 and miR-133 are transcriptionally induced by MRFs in cultured myoblasts after the switch to differentiation conditions, whereas miR-206 is already present in proliferating myoblasts prior to the onset of differentiation, when it is further induced by MRFs [50,59]. Conversely, in post-natal mature myofibers, miR-1 continues to be present whereas miR-206 expression appears undetectable suggesting that miR-1, and not miR-206, could be involved in the homeostasis of differentiated muscle, as already suggested in flies [48,50,52,61].

The myogenic role of myomiRs is sustained by a reciprocal direct and indirect regulation of MRFs expression (Table 2). miR-1 directly targets MEF2 regulating neuromuscular synapse function in worms [62] and, like miR-206, is able to repress hystone deacetylase 4 (HDAC4) preventing the down-regulation of MEF2 and the inhibition of cell differentiation in myoblasts (Table 2) [49,63-65]. Additionally, both miR-1 and miR-206 prevent the expression of *Paired box 7 *(*Pax7*) and *Pax3*, both inducers of proliferation in satellite cells and myogenic precursors [66-68]. In turn, Pax7 can modulate miR-206 expression inducing the HLH inhibitor of differentiation Id2 that restrains MyoD activity [67].

**Table 2:** miRNAs and their target genes validated in myoblasts and/or in rhabdomyosarcoma cells

myomiRs	Target gene	Refs
miR-1	***cMet*** ***HDAC4*** *PAX3* *PAX7* *Hand2* *Mef2*	[77,78] [41,55,58] [61] [59] [50,62] [55]
miR-133a-1/133a-2	*SRF* *Cyclin D2*	[42]; [63]
miR-206	***cMet*** ***HDAC4*** *DNA-polα* *PAX3* *PAX7* *Connexin43* *Fst1*	[77,78] [41,80,57] [42] [61] [59,60] [45,42] [44]

**Non-muscle miRNAs**		

miR-29b2/miR29c	***YY1*** ***HDAC4***	[67] [80]
miR-26a	*Ezh2*	[66]
miR-183	***EGR1***	[86]

In **bold **are target genes identified in rhabdomyosarcoma cells.

In addition to these synergistic effects, each specific miRNA within the miR-1/miR-206 family can function differently due to their specific seed sequences that target different mRNAs even in the same tissue and conditions. For instance, miR-1 can inhibit cardiac myocyte growth by targeting the Hand2 transcription factor mRNA [57,69]. miR-206 down-regulates DNA pol α favoring cell cycle arrest and follistatin to amplify pro-myogenic signal [50,52]. Moreover, miR-1 and miR-206 can induce muscle cell differentiation, while miR-133, when artificially expressed in myoblasts, decreases the expression of myogenin and the late muscle marker myosin-heavy chain (MHC) and promotes proliferation [49,70]. This effect seems to be related to miR-133-mediated down-regulation of SRF, which is a weak activator of muscle-specific genes and regulates the balance between proliferation and differentiation [49]. Therefore, miR-133 can sustain one or the other process depending on SRF availability and activity and SRF downstream cofactors present in a cell timing and context.

The diversity in myomiRs function and pattern of expression in muscle tissues can be also explained by the fact that they can be differentially and independently induced through their own muscle-specific promoters and enhancers (Figure 2) [71]. Therefore, the availability of each specific muscle regulatory factor and the accessibility of gene regulatory sites drive strictly controlled tissue specific miRNA expression.

Taken together, these observations are consistent with differential global gene expression profiles induced by each type of myomiR in a context-dependent fashion. This variety of similar, different or even opposite effects of specific myomiRs provides molecular support for proper muscle tissue development highlighting the complexity of miRNAs function.

#### Non-muscle-specific miRNAs in skeletal muscle differentiation

Some miRNAs that are also expressed in other tissues have been shown to play a role in vertebrate muscles [72-77]. All these miRNAs promote myogenesis by impairing the proliferation of muscle cell precursors through the down-regulation of genes that repress muscle differentiation. Among these miRNAs, miR-181, miR-27, miR-26a and miR-29b2/miR-29c have been shown to be deregulated in RMS. During myogenesis, the expression of the miR-181a/miR181b cluster is strongly up-regulated and positively acts in tissue determination by inhibiting the expression of homeobox gene *HoxA11*, which is an inhibitor of terminal muscle differentiation [75]. At the onset of myogenesis, the *Pax3 *3'UTR is targeted by both miR-27a and miR-27b, encoded by genes on different chromosomes. This induces a shift of Pax3-positive cells to Myogenin-positive cells [73]. Recently, several lines of evidence indicate a role for miRNAs as regulators of epigenetic processes during tissue differentiation. miR-26a promotes myogenesis by targeting the mRNA of histone methyltransferase *Enhancer of zeste homolog 2 *(*Ezh2*) [77,78]. EZH2 is a Polycomb group (PcG) protein that catalyzes the trimethylation of lysine 27 on histone H3 of target gene promoters. Acting as a part of the Polycomb Repressive Complex 2 (PRC2), EZH2 contributes in maintaining repressive chromatin structures that inhibit the transcription of key developmental genes [79,80]. In satellite cells and myoblasts undergoing differentiation, EZH2 inhibits myogenesis by directly repressing the transcription of late-stage muscle-specific genes such as MHC and muscle creatine kinase (MCK) [81]. Interestingly, in murine satellite cells that are in specific differentiation stages, inflammatory conditions induce isoform-specific p38-dependent EZH2 phosphorylation that results in the repression of Pax7 promoter, impairing the expansion of muscle progenitors [82].

EZH2 may be a key target gene of other non-muscle-restricted miRNAs induced by MRFs [83] but not investigated in the RMS context, such as miR-214, which is maintained in a repressive state by EZH2 as a part of a regulatory feedback loop prior to the onset of differentiation [78]. It is noteworthy that the timing of expression of miR-26a and miR-214 differs during myogenesis. miR-214 is up-regulated early, preceding p21^Cip1 ^and myogenin expression [78,83], whereas miR-26a expression increases more gradually during the course of myogenesis [77]. Although ectopic over-expression of miR-26a is sufficient to trigger myoblast differentiation in parallel with EZH2 down-regulation, it is possible that, during physiological development, miR-26a could act by reinforcing, rather than triggering, myogenesis.

**Figure 3:**
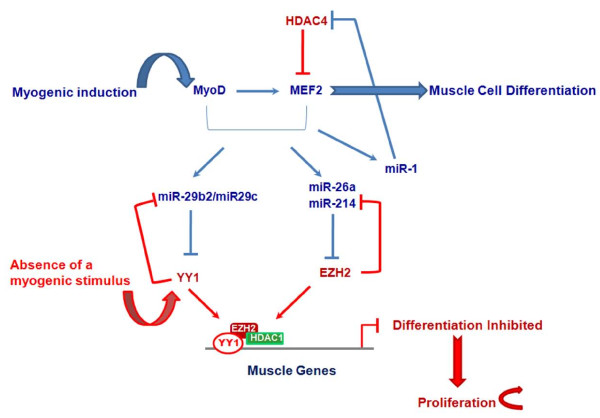
**Model for circuits involving Polycomb Group (PcG) proteins and miRNAs during muscle cell differentiation**. In blue: in differentiating myoblasts, miR-29b2/miR-29c, miR-214 and miR-26a are induced by muscle-specific transcription factors, such as MyoD and MEF2, and post-transcriptionally block the expression of YY1 and EZH2 PcG proteins. Together with the inhibition of HDAC4 expression by miR-1, these phenomena lead to differentiation of myogenic precursor cells. In red: in the absence of a differentiative stimulus, YY1 and EZH2 are highly expressed and foster the proliferation (*i.e*., expansion) of progenitor cells by repressing the expression of miR-29 and miR-214 and down-regulating muscle-specific genes.

Recently, the group of Guttridge has shown that the miR-29b2/miR-29c cluster is a target of the PcG transcription factor Yin Yang 1 (YY1), which is induced in an NF-kB-dependent manner in the absence of a myogenic cue [76]. Authors showed that, besides myofibrillar genes [81,84], YY1 represses miR-29b2/miR-29c transcription by recruiting EZH2 and HDAC1 on its promoter (Figure 3). This process results in the expansion of undifferentiated muscle precursors. Consistent with a model of miRNA-dependent suppression of the epigenetic control during myogenesis, in response to myogenic program activation, miR-29b2/miR-29c begins to be expressed and inhibits the expression of YY1, thus accelerating skeletal muscle differentiation [76]. Interestingly, although no direct targeting of miR-29b2/miR-29c on the 3'UTR of EZH2 was detected, EZH2 levels decrease after forced re-expression of miR-29b2/miR-29c. This suggests indirect mechanisms induced by this miRNA, and possibly other miRNAs acting on epigenetic mediators, to regulate epigenetic pathways as a whole. Moreover, NF-kB loss-of-function experiments in myoblasts demonstrate that both YY1 and EZH2 are unable to bind the enhancers on miR-29b2/miR-29c promoters in the absence of an activated NF-kB signal. This observation supports the hypothesis that the NF-kB pathway regulates this YY1-EZH2/miR-29b2/miR-29c network. Therefore, epigenetic molecular networks involving feedback regulatory loops with miRNAs may play a key role in myogenesis.

### Muscle-specific miRNAs in RMS

Most studies on the involvement of miRNAs in RMS pathogenesis and their potential therapeutic uses in RMS have been conducted on the myomiR family miR-1/miR-206. We and others have shown that the expression of miR-1 and miR-133a is strikingly decreased in alveolar and embryonal RMS cell lines compared to differentiated myoblasts and skeletal muscle tissues [85-88]. In particular, two pre-clinical studies reported that forced re-expression of miR-206 leads to cell cycle arrest and myogenic differentiation of RMS cells, preventing xenografts growth *in vivo *by targeting the mRNA of the oncogenic c-Met receptor [86,87]. The Ponzetto group demonstrated that miR-1 and miR-206 are down-regulated in both alveolar and embryonal RMS compared to non-neoplastic skeletal muscle tissues, and that they fail to increase in RMS cell lines in response to differentiation-inducing treatment [86]. Moreover, re-expression of miR-1 or miR-206 through lentiviral vectors promotes cell differentiation also in alveolar cell lines that are quite resistant to differentiative cues, and blocks anchorage-independent growth and invasiveness *in vitro*. Elegant studies with inducible lentiviral vectors expressing miR-206 at different times after RMS xenografts implantation *in vivo*, clearly demonstrated that re-expression of miR-206 prevents tumor growth [86]. Finally, clusters of hundreds of genes up- (muscle lineage) or down-regulated (cell cycle) by miR-206 in RMS were identified, among which c-Met was shown to be a miR-206 direct target. The miR-206-dependent post-transcriptional inhibition of c-Met expression markedly contributes to the anti-tumor effects of this miRNA.

Similar results are reported in a manuscript published almost simultaneously to that of Taulli et al. [86] on an embryonal RMS cell line [87]. This study too showed a down-regulation of miR-1 and miR-206 in RMS primary samples compared to normal muscles, and reported that forced expression of either miR-1 or miR-206 in the embryonal RMS cell line RD *in vitro *and *in vivo *blocks its tumorigenic potential. Consistent with data from Ponzetto's group, these phenomena occur through miR-206 direct targeting of c-Met mRNA.

More recently, Rao et al. [88] showed that miR-1 forced expression in the RD cell line promotes muscle gene expression and cell cycle arrest, while miR-133a leads to a decrease of muscle markers expression. This is consistent with different roles of miR-1 and miR-133 in normal muscle differentiation. However, in contrast to what occurs in healthy myoblasts, both miRNAs inhibit cell growth in RMS cell lines. This finding highlights, once more, the importance of cell context in determining the response to miRNAs modulation.

The clinical potential of re-expression of miR-1/miR-206 clusters in RMS is further supported by the observation that these miRNAs directly regulate HDAC4 during differentiation. This is of great importance because, among other effects, HDAC4 is responsible for preventing the expression of cyclin-dependent kinase inhibitor p21^Cip1 ^that is essential for muscle differentiation [89,90]. To date, HDAC inhibitors appear as promising agents for targeted treatment of metastatic RMS [91]. However, re-expression of miR-1/miR-206 clusters is likely to have more complex effects than HDAC4 silencing and may be therapeutically more effective [28].

### Non-muscle-specific miRNAs in RMS

**Figure 4:**
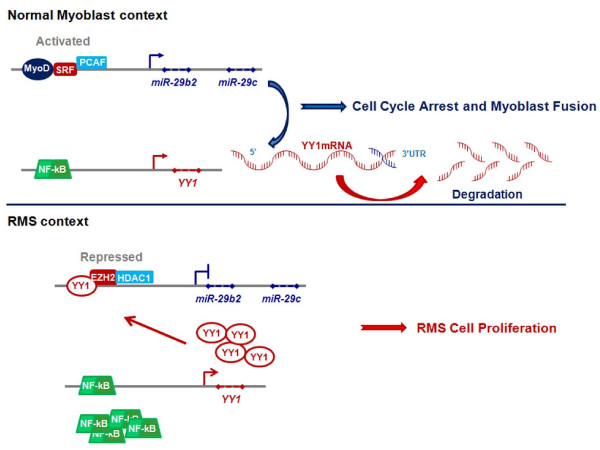
**Dysregulation of miR-29/YY1 circuit in rhabdomyosarcoma cells**. Upper panel, During muscle tissue formation, in normal myogenic precursor cells miR-29b2/miR-29c targets the 3'UTR of YY1 mRNA inhibiting its expression. **Lower panel**, Conversely, in rhabdomyosarcoma (RMS) cells NF-kB is up-regulated and YY1 over-produced. The high amount of YY1 in RMS cells is able to recruit EZH2 and HDAC1 on the promoter of miR-29 gene blocking its transcription thus resulting in uncontrolled cell proliferation.

Recently, de-regulation of miR-29 has been reported in a small cohort of alveolar RMS [92]. A role of the miR-29b2/miR-29c cluster in RMS pathogenesis has been confirmed by the recent study of Wang et al. [76]. Previously, these authors showed that an NF-kB-dependent pathway necessary for the expansion of undifferentiated myogenic precursors, is aberrantly activated in RMS cells [84]. In their latest study, they showed that NF-kB activation in RMS leads to over-expression of YY1 which interacts with EZH2, causing sustained down-regulation of miR-29b2/miR-29c and repression of myogenesis (Figure 4). Consistent with an anti-myogenic role of these two PcG proteins, their levels were found up-regulated in tumor tissues from RMS patients compared to normal adjacent muscle tissues [76]. Interestingly, to repress miR-29b2/miR-29c expression in RMS cells, YY1 recruits EZH2 to a different site of the miR-29b2/miR-29c promoter than the one used during the expansion of normal myoblasts. Ectopic expression of exogenous miR-29b2/miR-29c leads to cell cycle arrest and differentiation of RMS cell lines, and inhibits RMS xenograft growth. Consistent with this observation, miR-29b2/miR-29c levels have been shown to be reduced in tumor samples compared to control muscle tissues. This study was the first to suggest a potential "differentiation therapy" of RMS through re-expression of a pro-myogenic miRNA that is involved in the epigenetic control of differentiation. Along the same lines, our group showed that EZH2 expression is increased in tumor tissues from RMS patients independently of histological subtype, and correlates with markers of poor prognosis (Abstract # 10-A-4051 AACR 2010). Studies on a larger cohort are underway to determine whether the level of EZH2 expression correlates with the presence of fusion proteins.

In addition, we showed concomitant abnormal expression of miR-26a and EZH2, the former being highly down-regulated and the latter abnormally expressed in RMS tumor samples and cell lines compared to controls [85]. However, the role of miR-26a in restoring epigenetic processes in RMS needs to be fully elucidated.

Interestingly, miR-29b has recently been shown to directly target HDAC4 during osteoblast differentiation suggesting that this aspect of miR-29b-dependent regulation could be also involved in muscle tissue differentiation and possibly in RMS pathogenesis [93]. A further role of miR-29 in epigenetic regulation has been highlighted by studies on lung cancer showing that this miRNA targets DNA methyltransferases, leading to a global down-regulation of DNA methylation when re-expressed in tumor cells [94]. Notably, an interconnection among miR-29 and miR-206 has been unveiled in liver [95]. These authors showed that miR-206 is repressed by a YY1/AP1 complex on its promoter, and YY1 down-regulation leads to miR-206 de-repression. This suggests a rationale for future investigations of this process in muscle tissues and RMS [95].

A recent publication describes high levels of miR-183 in RMS cell lines and primary tumors [96]. This miRNA behaves as an onco-miR in several cancers and it has not been previously associated with muscle. The miR-183 pro-tumorigenic role in RMS is supported by the evidence that tumor cells in which this miRNA is knocked-down show reduced cell migration *in vitro *[96]. This phenomenon is due to the release of the direct repression of *Early growth response 1 *(*EGR1*), a regulator of cell migration, by miR-183. Anti-miR-183 treatment stimulates the expression of the tumor suppressor gene Phosphatase and tensin homolog (*PTEN*) as well that, in turn, fosters EGR1 expression reinforcing the inhibition of cell migration.

Finally, our group has shown that miR-27a is significantly down-regulated in RMS tissues and cell lines, especially in the alveolar subtype [85]. This is consistent with its pro-myogenic role in normal development [73]

Taken together, these data underscore the complexity of miRNA function and regulation in RMS and their central role in modulating the transition between a differentiative *versus *an activated cell state. Moreover, the data we reviewed point to the fact that apparently minor changes in gene expression, even only in one miRNA, could affect the delicate balance between physiologic and pathologic cell fate programs.

### miRNAs as diagnostic and prognostic tools in RMS

One of the first studies on miRNAs expression in the clinical context of RMS focused on amplification of the 13q31-32 chromosomal region, which is amplified in a fraction of alveolar RMS patients [97] and includes the *C13orf25 *gene [98]. This gene contains the miR-17-92 cluster (miR-17, miR-19a, miR-19b, miR-20a, and miR-92), which is considered an onco-miR cluster in some tumor types and cross-talks with *MYC*, an oncogene amplified in about 20% of fusion-positive alveolar RMS. This study shows that miR-17-92 expression did not correlate with *C13orf25 *gene amplification in all RMS samples, irrespective of their alveolar or embryonal origin, suggesting that mechanisms other than amplification could be responsible for miRNA over-expression.

More recently, the Barr group [99] investigated a minimal common region of the 13q31 amplicon that contains the miR-17-92 cluster gene in alveolar RMS. These authors showed that the 13q31 amplification was present in about 23% of alveolar RMS, preferentially in *PAX7-FKHR*-positive cases compared to *PAX3-FKHR*-positive and fusion-negative tumors. The majority of alveolar RMS amplified for 13q31 expressed high levels of five out of six miRNAs within the miR-17-92 cluster, except for miR-18a. Unexpectedly, also a group of tumors that lack 13q31 amplification showed high expression of all six miRNAs in the miR-17-92 cluster, although the level of expression was lower than in amplified cases. This finding supports the idea that multiple mechanisms in addition to gene amplification regulate miR-17-92 expression in RMS, as previously reported in primary tumors and cell lines [98,100]. Moreover, it suggests that the expression of the entire miRNA cluster can be controlled by a common regulatory mechanism. Notably, high levels of the five miRNAs in the 13q31 amplified group of patients, most of whom were *PAX7-FKHR*-positive, were directly and independently correlated to a worse outcome when compared to non-amplified cases. Interestingly, expression levels of these miRNAs were inversely correlated with outcome within the amplified RMS group. Therefore, although further studies are needed to identify the molecular basis for these correlations, collectively these results associate amplification and expression of the miR-17-92 cluster with specific subsets of alveolar RMS and could be useful as prognostic biomarker in these tumor forms.

The clinical relevance of the dysregulation of the miR-1/miR-206 family has been recently highlighted by a study on a large cohort of 163 RMS patients [101]. Besides confirming that all these miRNAs are down-regulated in RMS samples compared to muscle controls, the study of Missiaglia et al. [101] shows that alveolar RMS specimens positive for fusion proteins PAX3- and/or PAX7-FKHR have higher miR-1 levels compared to fusion gene-negative samples. This is an interesting finding, considering that alveolar RMS cells usually express higher amounts of myogenic factors than embryonal ones and that high level of myogenin expression has been recognized as a biomarker of adverse prognosis in RMS [102,103]. Important from a clinical/translational standpoint, these authors identified an inverse correlation between the expression of miR-206 and overall survival within both the whole RMS group and the gene fusion-negative subgroup of patients, while no correlation was observed for gene fusion-positive samples. Additionally, miR-206 was shown to be lower in patients with advanced stage-disease and metastasis at diagnosis, even though significant correlations were detected only for fusion gene-negative patients. These findings highlight the potential of miR-206 expression as a marker of prognosis and disease progression, especially in embryonal tumors that lack specific biomarkers of aggressiveness. Consistently with the role of miR-206 in muscle determination, gene expression analysis showed that markers of differentiation are positively correlated with miR-206 expression in RMS samples. Interestingly, the expression of inflammatory molecules was inversely correlated with that of miR-206 suggesting that miR-206 could be down-regulated by inflammatory networks in RMS, as already shown for miR-29b2/miR-29c [76,101]. In contrast, miR1 and miR-133 do not show any correlation with the survival probability in patients.

Recently, the expression levels of a specific miRNA signature were reported to classify RMS patients into 4 subgroups, *i.e*., PAX3-FKHR, PAX7-FKHR and fusion-negative alveolar RMS and embryonal RMS [45]. Although the cohort of patients was small, this result is of particular interest since it suggests that miRNAs expression could be helpful in classifying RMS discriminating between alveolar fusion-negative and embryonal RMS that are often molecularly indistinguishable with current techniques.

The evidence that miRNAs can be released, via different mechanisms, in human peripheral blood and their relative stability and consistent levels in circulation has suggested that they can be used as non-invasive biomarkers [104-107]. Among the myomiRs, miR-206 is the most tissue-representative, as it is expressed almost exclusively by skeletal muscle during development and regeneration and is almost undetectable in adult normal skeletal muscle. On this basis, miR-206 circulating levels have been investigated in sera of RMS patients and found to be higher as compared with sera from healthy donors or from pediatric patients with other tumors [108]. Considering that miR-206 levels are inversely correlated with good prognosis, the possibility to detect its presence in serum of patients could help in the follow-up of highly aggressive neoplasms. This might open the way to a non-invasive approach to the diagnosis and follow-up of RMS, which could facilitate the rapid implementation of aggressive treatment protocols and improve prognosis [109,110]. A possible drawback may be related to the expression of muscle-specific miRNAs in extremely rare cases of myogenic tumors of childhood such as leiomyosarcoma and rhabdomyoma. Concerning the use of miRNA markers in clinical practice, it must be considered that miRNAs quantification methods in body fluids are still under development, due in part to the small amounts of circulating miRNAs, especially in serum *vs *plasma [111,112]. In addition, the choice of an endogenous control remains critical since no housekeeping miRNAs have been identified so far [104,113,114]. Despite the need for more studies to standardize the measurement methods [113], results reported by Miyachi and co-workers appear promising [108].

Finally, miRNAs have been recently hypothesized to regulate drug responsiveness [115,116]. A direct link between miRNAs and drug responsiveness of RMS cells has been recently unveiled by a study demonstrating that down-regulation of miR-485-3p is responsible for the Nuclear Factor- (NF)-YB-dependent decrease in DNA Topoisomerase II (Top2) in the etoposide-resistant RH30/v1 RMS cell line [117]. The transcription factor NF-YB binds the *Top2 *gene promoter, inhibiting its transcription and thus reducing the effect of Top2 inhibitors. Re-expression of miR-485-3p in RH30/v1 cells reduces NF-YB levels and restores Top2 expression. These effects are associated with an increase in sensitivity of RMS cells to Top2 inhibitors *in vitro*. This discovery could shed light on one of the mechanisms of drug resistance to Top2 inhibitors in this soft-tissue sarcoma and suggest new therapeutic opportunities and pharmacodynamic biomarkers. However, several technical problems such as the choice of a good control miRNA for normalization and standardization of procedures [114,118], will need to be solved before miRNAs detection in clinical in samples can have practical applications.

### Perspectives and conclusions

In summary, recent studies on miRNAs have shown that miRNA expression underlies a complex layer of gene regulation events guiding biological processes that are fundamental for tissue-specificity and homeostasis [28]. Some miRNAs that participate in skeletal/cardiac muscle tissue determination have been identified. It is conceivable that, in the future, more miRNAs will be discovered that are potentially able to re-establish correct differentiation in RMS through the modulation of diverse molecular pathways.

Although the re-expression of selected miRNAs is a possible strategy for targeted therapy in RMS, it must be noted that miRNA-based therapy presents several challenges. Selectively targeted, efficient re-expression of miRNAs is the primary need for an effective therapy. To date, viral and non-viral vectors have been used in pre-clinical studies to deliver miRNAs. However, viral vectors, though efficient in the expression of cDNAs, can be limited in their practical applications by immunogenicity and lack of specificity. Non-viral cationic liposome-mediated gene transfer approach could be attractive for miRNA therapy; however, cationic liposomes developed so far suffer from low efficiency of cell transduction. Moreover, the instability of miRNAs *in vivo *and the potential immunostimulatory effects of double-stranded RNAs are serious obstacles to therapy based on direct delivery of miRNAs. Recently, several types of nanoparticles have been proposed as an alternate, highly efficient vehicle to deliver DNA particles to cancer cells [119] and they have been used in preclinical studies for an RNA-interference therapy [120]. More recently, two studies have shown that anti-miRNAs molecules stabilized in complexes with either lysine-containing or vessel-targeted nanoparticles are capable to decrease the expression of a liver-specific miRNA or that of a pro-angiogenic miRNA when systemically delivered *in vivo *[121,122].

Recently, locked nucleic acid (LNA) oligos anti-miRNA were evaluated in non-human primates with unexpectedly positive results [123,124]. Altogether, these results appear encouraging for a possible inhibitory approach using anti-miRNAs against onco-miRs.

In recent years, "epigenetic" therapies aiming at modulating gene expression at the transcriptional level have attracted increasing attention. Such treatments have given promising results in clinical trials for some types of tumors [125-127]. In addition to well-known epigenetic drugs acting as either DNA-demethylating agents or HDAC inhibitors, researchers are working on a class of agents that inhibit histone methyltransferases such as EZH2, and do not require cell division to target cancer cells [128,129]. Interestingly, histone methyltransferase inhibitors have been shown to synergize with other epigenetic agents in preclinical studies [130-132].

Since EZH2 negatively regulates the expression of pro-myogenic miRNAs, such as miR-214 and miR-29b2/miR-29c, histone methyltransferase inhibitors may be able to restore physiological levels of expression for these miRNAs in RMS. Thus, the use of more traditional pharmacological agents could overcome the delivery problems associated with "gene therapy" approaches. On the other hand, "epigenetic" drugs can affect a variety of molecular networks and their *in vivo *mechanism of action remains controversial.

It is noteworthy that miRNA expression can be regulated by epigenetic modifications *per se *such as DNA methylation or histone acethylation [23]. Indeed, approximately 50% of miRNA genomic sequences are associated with DNA regions subjected to methylation, such as CpG islands, and thus are often methylated in cancers resulting in silencing of tumor suppressor miRNAs [133]. Conversely, hypomethylation of miRNA genes that can lead to over-expression of oncogenic miRNAs can contribute to tumorigenesis [134,135]. Moreover, the same onco-miR can be hypomethylated or hypermethylated depending of the specific tumor context, suggesting a tissue type-dependent epigenetic regulation [135,136]. Therefore, an epigenetic therapy would have to be carefully studied, since it could induce the re-expression of oncogenic molecules. This has been the case with some HDACs and DNA methylation inhibitors that have been recently reported to increase the metastatic capability of xenografted tumor cells in an animal model of RMS through the de-repression of the pro-metastatic *Ezrin *gene [137]. Interestingly, the Subramanian group [96] reports that miR-183 silencing in RMS cells is associated with a lowering of Ezrin levels. This report suggests that, besides the re-expression of pro-differentiative miRNAs, a concomitant inhibition of onco-miRs may be valuable in combination with an epigenetic therapy.

The high number of mRNAs targeted by a single miRNA may represent an advantage compared to specific gene silencing (e.g., siRNA). However, this also means that each miRNA can modulate several molecules/pathways with potentially unpredictable side effects. Therefore, miRNA expression should be controlled with the aim to achieve physiological levels rather than overexpressing miRNAs. A more detailed understanding of molecular events governing myogenesis is needed for the identification of myogenic functional steps and networks in which these small RNAs participate. Nonetheless, the potential of a therapy based on re-expression of tumor suppressor miRNAs in RMS is high, considering that miRNA re-expression has been shown to overcome drug resistance in several types of tumor cells and in RMS cells *in vitro *[117,138-140]. A "miRNA therapy" may be used in the future in combination with conventional therapy in high-risk RMS patients with metastatic disease, often refractory to conventional therapy. Moreover, miRNA expression profiling in tumors, and possibly, their detection in peripheral blood during treatment, could predict the response to chemo- and/or radiotherapy and be useful as a prognostic signature for the development of treatment resistance.

## Competing interests

The authors declare that they have no competing interests

## Authors' contributions

RR selected the literature, wrote the manuscript and reviewed the final version. RC contributed to the conception of the manuscript and to critical discussion. FL, AG and LM contributed to the discussion on clinical implications and reviewed the manuscript. All authors read and approved the final manuscript.

## Authors' informations

RR is a PhD and the Head of the Laboratory of Angiogenesis with experience in mechanisms that regulate gene expression and cell growth in pediatric cancers. RC is a PhD working on transcriptional regulation in cancer in the Laboratory of Angiogenesis directed by RR. FL is an MD and Full Professor of Pediatrics and the Head of the Oncohematology Department with a long standing experience in preclinical research and clinical management of pediatric tumor patients. LM is an MD and the Director of Cancer Centre and Professor of Medicine and Pharmacology at the University of Mississippi Medical Center in Jackson, MS, who has a long experience in preclinical and clinical research targeting developmental and cell fate pathways in solid tumors, particularly breast cancer. AG is an MD and Full Professor in Pathology with long lasting experience in the study of gene expression and cell cycle regulation in cancer.
